# Regiochemical Control of Shape Morphing in Diels–Alder Covalent Adaptable Networks

**DOI:** 10.1021/acsmacrolett.5c00465

**Published:** 2025-10-01

**Authors:** Yilei Zhao, Junho Moon, Svetlana A. Sukhishvili

**Affiliations:** Department of Materials Science and Engineering, 14736 Texas A&M University, College Station, Texas 77843, United States

## Abstract

We demonstrate that regioisomerism in Diels–Alder (DA) reactions offers a subtle yet powerful way to tune the thermomechanical and shape morphing behavior of dynamic polymer networks. Here, we directly compare DA polymer (DAP) networks built from linear polymers with identical backbones but containing either 2- or 3-substituted furan pendant groups. For a wide range of cross-linking degrees, the 3-substituted DAP (3-DAP) networks exhibited higher thermal stability, with retro-DA dissociation temperatures (*T*_*rDA*_) of ∼150 °C versus 120 °C for the 2-substituted counterparts, higher elastic moduli and significantly slower temperature-dependent stress relaxation rates. The difference in the stress relaxation rates of 2- and 3-DAP elastomers was leveraged to demonstrate controlled bending in a bilayer structure via selective network plasticization. Moreover, due to the higher *T*_*rDA*_ values, the use of 3-DAP elastomers in the single-material constructs opened a wider and a higher-temperature window (i.e., between 80 and 140 °C) for shape morphing, which is more practical compared to the traditionally employed DA-based networks such as 2-DAP elastomers which could be morphed only between 60 and 90 °C. These findings establish regioisomerism in the furan ring as a powerful parameter for designing DA-based shape morphing materials.

## Graphical abstract

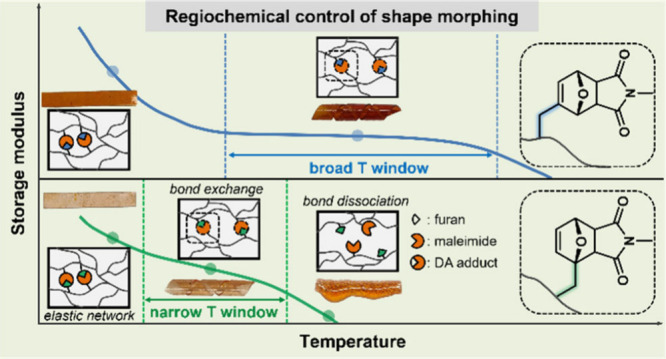

Covalent adaptable networks (CANs) bridge the gap between the traditional thermoplastics and thermosets,^1–3^ enabling a unique combination of materials’ behaviors, such as adaptation to the environment,^4,5^ self-healing^6–10^ and reprocessability.^11–13^ One important type of CANs is based on the reversible Diels–Alder (DA) “click” reactions^14–17^ between furan and maleimide which can be partially dissociated at mild temperatures, enabling reconfiguration of the materials’ shape.^18–21^ Recently, we have explored the role of stereochemistry (i.e., contributions of *endo vs exo* DA adducts) in shape morphing of DA polymer (DAP) networks.^22^ However, the role of regiochemistry, i.e., substitution in the furan ring, in reconfigurability of CAN networks remained unexplored, as current covalent dynamic networks are typically constructed using 2-alkyl-substituted furan moieties.^23–25^

Yet, the effects of substituents in the furan ring on kinetics and thermodynamics of DA reactions have been addressed theoretically and studied in solutions of small molecules.^26–29^ These studies indicated that due to the differences in electron density distribution in the furan ring and steric constraints at the reactive sites, 3-substituted furans form DA adducts with higher thermal stability compared to their 2-substituted counterparts.^26,27^ However, regiochemistry in conjunction with DA reactions is rarely explored for macromolecules, and the few known examples refer to solutions.^30,31^ In one study, the effect of regiochemical substituents in the furan ring was found to strongly influence the rate of the retro-Diels–Alder (rDA) reaction when DA polymers were activated in solution by ultrasound-generated elongational forces.^30^ Another study demonstrated that regioisomerism influences mechanical strength and resistance to force-induced bond dissociation in hydrogels.^31^ More recently, Wang et al. introduced 3-substituted furans into rigid epoxy-based polymers to leverage the cross-links for enhancing material’s thermal stability and stiffness.^32^ However, the rigid and brittle polymers explored in the latter study do not allow exploiting the dynamicity of CANs for shape morphing, which typically requires the elastomeric state to enable large deformations.

Here, we explore the impact of regiochemistry in the DA reaction on the thermomechanical and shape morphing properties of dynamic covalent networks. Through a side-by-side comparison of CANs constructed from 2- and 3-substituted furans (2-DAP and 3-DAP, respectively), we systematically tune materials’ softness via the number of DA cross-links and investigate how differences in furan ring regiochemistry influence the resulting thermomechanical properties. We then focus on softer, less cross-linked networks to evaluate the reconfigurability of 2-DAP and 3-DAP materials. Building on our previous studies which demonstrated that stereochemical variations (*endo*/*exo* isomers) in DA polymer networks can be leveraged for programmable shape morphing,^22,33^ this work shifts the focus from stereoisomerism to regioisomerism, uncovering how differences in furan substituent position can be harnessed to develop new design routes for materials’ shape morphing.

To investigate how isomerism in the furan ring affects network formation and thermomechanical behavior, we synthesized dynamic polymer networks using epoxy prepolymers with matched molecular parameters that differed only in the type of the substituent (2- vs 3-substituted) in the pendant furan rings (Figure 1a). The prepolymer used for 2-DAP networks was synthesized as described in our previous work,^16,17,21,22,33^ while synthesis of the prepolymer used for 3-DAP networks was similar (see Supporting Information) but involved temperature as an additional parameter to control the prepolymer molecular weight (Figure S1a). Both prepolymers used for CAN synthesis were prepared at a 1:1.9 ratio of the amine-to-epoxide groups, resulting in the weight-average molecular weights (*M*_*w*_) of ∼7 kDa and *T*_*g*_ of −14 to −15 °C (Figures S1b,c). The network formation was initiated by introducing 1,1′-(methylenedi-4,1-phenylene)­bismaleimide (BMI) into each prepolymer. The network thermomechanical properties were additionally tuned by stoichiometry, specifically the molar ratio of the maleimide-to-furan groups, Φ_BMI_.^21^ Networks prepared with varying Φ_BMI_ controlled by the amount of BMI cross-linker are denoted as DAP Φ_BMI_, where Φ_BMI_ ranged from 0.2 to 1.0. To further assess whether Φ_BMI_ directly correlates to effective cross-link density, we plotted the rubbery-plateau storage modulus (*E*_*plateau*_^′^) as a function of Φ_BMI_ (Figure S2). The linear relationship between *E*_*plateau*_^′^ and Φ_BMI_ indicates that the number of elastically active cross-links increased proportionally to BMI content, suggesting high efficiency of cross-linking.

**Figure 1:**
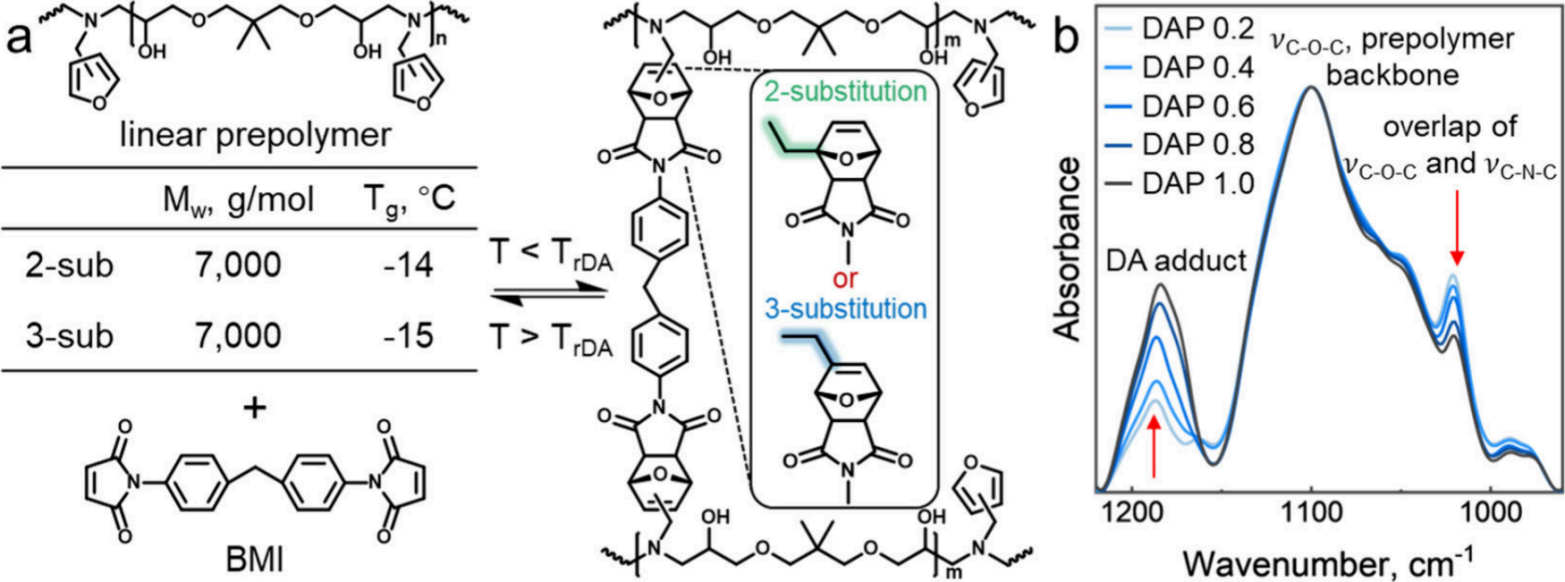
Synthesis and spectroscopic analysis of DAP networks built with 2- and 3-substituted furans. (a) Schematic representation of formation and temperature-triggered dissociation of 2- and 3-DAP networks, with the embedded table showing the weight-average molecular weights (*M*_*w*_) and *T*_*g*_ of the corresponding prepolymers. (b) ATR-FTIR spectra of 3-DAP Φ_BMI_ networks with Φ_BMI_ varying from 0.2 to 1.0.

Due to the different thermodynamics of the DA reactions for the prepolymers with different regio-substituents,^34,35^ cross-linking of the prepolymers with BMI was performed in different conditions. Specifically, for the synthesis of 2-DAP networks, the BMI cross-linker was directly added to the liquid prepolymer heated at 120 °C, i.e., at the temperature of rDA reaction (*T*_*rDA*_) of the 2-furan/maleimide adducts, without the use of solvent. This enabled rapid and complete DA cross-linking and solidification upon cooling, as established in our previous work.^17,21,22,33^ However, due to the higher thermal stability of the 3-substituted DA adduct (*T*_*rDA*_ ∼ 150 °C, Figure 2a) and the onset of BMI self-polymerization at >160 °C,^36,37^ this protocol could not be followed and 3-DAP networks were prepared via a solvent-assisted mixing at mild temperatures, followed by solvent evaporation (see Supporting Information). This route, which is also used by many groups for network synthesis,^38–40^ resulted in only partial cross-linking of as-synthesized networks and required additional thermal annealing at 60 °C to achieve DA reaction completion. Figure S3 in the Supporting Information shows that DA cross-linking in 3-DAP networks increased with time and limited off after annealing for 20 h at 60 °C or 12 days at room temperature as determined by the changes in glass transition temperature (*T*_*g*_). After annealing, conversion of DA reaction (i.e., the percentage of furan groups reacted with the added BMI cross-linker) was improved, as evidenced by the enhanced DA adduct peak intensity at 1190 cm^–1^ in the FTIR spectra^41^ (Figure S4).

**Figure 2:**
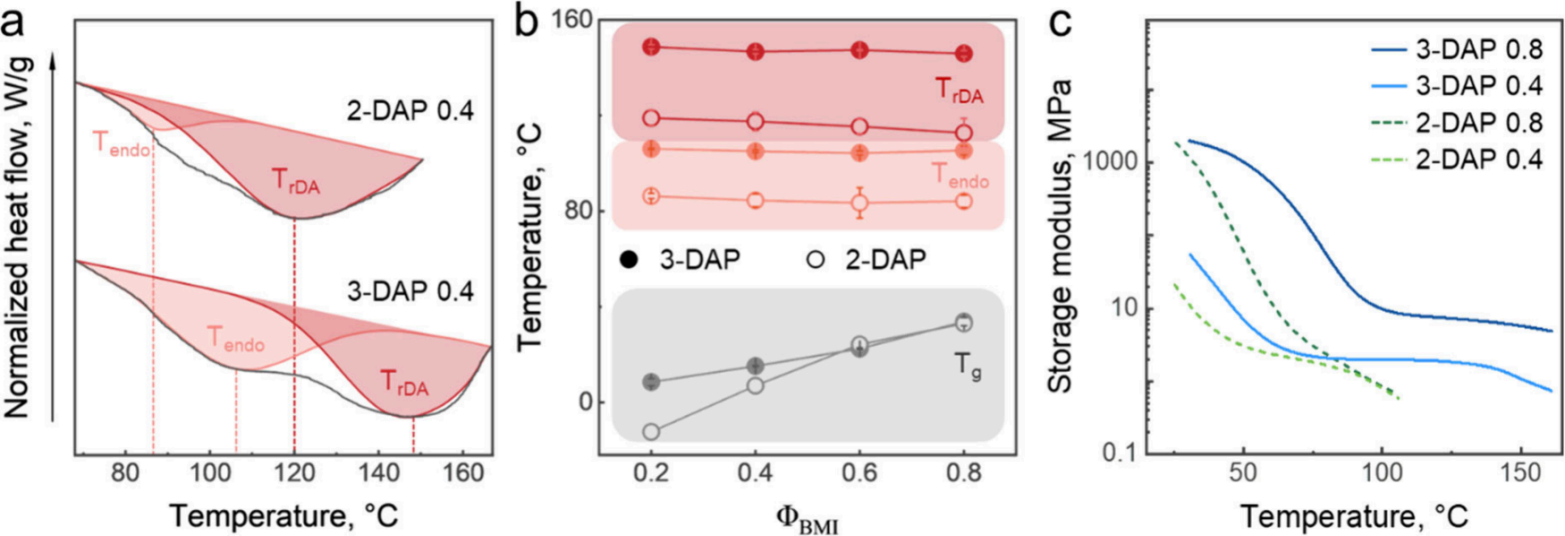
Thermal and thermomechanical properties of 2- and 3-DAP networks. (a) Differential scanning calorimetry (DSC) curves of 2-DAP 0.4 and 3-DAP 0.4 networks. *T*_*endo*_ and *T*_*rDA*_ were determined using deconvolution of the peak associated with the thermal transitions. (b) Comparison of *T*_*endo*_, *T*_*rDA*_, and *T*_g_ for 2- and 3-DAP networks with different Φ_BMI_, as determined by DSC. (c) Temperature dependence of storage modulus (*E’*) of DAP networks with Φ_BMI_ = 0.4 and 0.8 obtained via dynamic mechanical analysis (DMA).

All further experiments in this work were performed with 3-DAP networks in which conversion of furan groups to DA adducts was maximized via preannealing at 60 °C. Since the stereoisomeric composition (*endo*/*exo*) of DA adducts can also change during annealing and influence *T*_*g*_, we quantified the *endo* content as a function of annealing time (Figure S5). For the 3-DAP 0.4 network, only ∼5–6% *endo*-to-*exo* conversion occurred during the 4 h preannealing step required for complete DA bond formation, while *T*_*g*_ increased by ∼10 °C (Figure S3a). Continued annealing up to 10 h produced a further ∼5 °C increase in *T*_*g*_ which was accompanied by ∼10% loss in percentage of *endo* isomers, indicating that both conversion and isomerization contribute to the *T*_*g*_ increased. Nevertheless, under the 4 h preannealing used in this study, an increase in *T*_*g*_ was dominated by DA bond formation, while stereoisomeric changes remained limited (See Supporting Information for details).

Consumption of 3-substituted furan moieties due to the formation of DA cross-links in the networks with different Φ_BMI_ was monitored by FTIR. This analysis followed the same protocol as in our prior work on 2-DAP networks.^17,21,22,33^ As shown in Figure 1b for 3-DAP networks, the intensity of the peak at 1190 cm^–1^ associated with the −C–O–C– stretching vibrations in the DA adduct^41^ increased with maleimide-to-furan ratio, while the 1021 cm^–1^ peak, attributed to the overlap of −C–N–C– stretching vibrations in BMI and the unreacted furan, decreased due to the formation of the DA adduct. The analysis of FTIR data revealed that the extent of DA cross-linking scaled proportionally to the maleimide content, confirming that the cross-linking degree can be tuned by stoichiometry.

We then studied the effect of regioisomerism on the thermomechanical properties of CANs with widely varied Φ_BMI_. Figure 2a shows the distinct thermal behavior of 2-DAP and 3-DAP networks with Φ_BMI_ = 0.4 as a representative example. Both systems exhibited endothermal peaks indicating dissociation of kinetically controlled *endo* and thermodynamically controlled *exo* stereoisomers.^42^ The significant shift of both *endo* dissociation temperature (*T*_*endo*_) and the *exo* dissociation temperature (*T*_*exo*_ = *T*_*rDA*_) from ∼80 to 105 °C and from ∼120 to ∼150 °C for 3-DAP networks as compared to their 2-DAP counterparts is a clear indicator of a higher thermal stability of 3-substituted DA adducts. Figure 2b shows that the temperatures associated with the DA reactions (i.e., *T*_*endo*_ and *T*_*rDA*_) were increased with the cross-linking degree (i.e., Φ_BMI_ ranging from 0.2 to 0.8), suggesting that these temperatures represent the intrinsic thermodynamic properties of the DA bond formation. In contrast, *T*_*g*_ increased steadily as the networks became more densely cross-linked, due to the increasing constraints on the polymer chain mobility introduced by the cross-linking points. With an increase in cross-linking degree, both 2-DAP and 3-DAP networks transitioned from soft, elastomeric materials into rigid networks. At lower cross-linking degrees, 3-DAP exhibited a slightly higher *T*_*g*_ than 2-DAP networks with the same number of cross-links, possibly due to the additional interchain interactions such as hydrogen bonding between −OH groups and oxygen atoms in the unreacted furan, which can be sterically favored in 3-DAP networks. Similarly, 2-DAP and 3-DAP networks exhibited different mechanical properties. Specifically, Figure S6 shows that over a wide range of cross-linking degrees, 3-DAP networks shown consistently higher stiffness and ultimate tensile strength. For example, at a cross-linking degree of 40% (DAP 0.4 networks), 3-DAP showed an elastic modulus of ∼5.3 MPa—more than three times greater than that of 2-DAP (∼1.7 MPa) (Figure S6b).

The enhanced thermomechanical properties of 3-DAP materials were further evident from the temperature dependence of the network storage moduli (Figure 2c). Although the 2- and 3-DAP networks were prepared with the same cross-linking degree, they showed drastically different widths of the rubbery plateau. For 2-DAP, the storage modulus began to decline above 80 °C due to progressive dissociation of the network, ultimately leading to flow near its *T*_*rDA*_ of ∼120 °C.^21^ In contrast, 3-DAP networks maintained a much broader rubbery plateau up to ∼140–145 °C due to the higher *T*_*rDA*_ of the 3-substituted furan/maleimide adduct. The width of this plateau and the temperature ranges for the activation of dynamic bond exchange are the two key factors determining the usable temperature windows for shape reconfiguration. Here, we selected DAP 0.4 networks, as their lower cross-linking degree yielded elastomeric materials that are easily deformed, unlike the glassy and rigid networks obtained at higher cross-linking degrees. The temperature ranges for DA bond activation were probed in temperature-controlled stress relaxation experiments. Figure S7 shows that the activation of bond exchange in 3-DAP networks to achieve complete relaxation of the applied stress within ∼1 h required heating to 100 °C, while DA bonds in 2-DAP networks showed similar dynamics at a much lower temperature of 60 °C. In both cases, these temperatures were ∼60–70 °C lower than the corresponding *T*_*rDA*_ values of the DAP networks.

The differences in thermomechanical and stress relaxation behavior of 2- and 3-DAP networks were then leveraged to program materials’ shape-morphing. Figure 3a highlights the distinct stress relaxation rates for 2- and 3-DAP 0.4 networks at 60 °C under a 15% tensile strain. The 15% strain was chosen because it falls within the linear viscoelastic region of both materials (Figure S6b), ensuring that the relaxation reflects intrinsic network dynamics rather than nonlinear deformation. Under identical conditions, 2-DAP networks exhibited rapid stress relaxation within 30 min, while the 3-DAP network retained most of the applied stress throughout the test. This mismatch in stress relaxation was first leveraged to enable thermally induced shape morphing in a bilayer design. Bilayer constructs were prepared using ∼0.6 mm-thick 2-DAP and 3-DAP films with the matched cross-linking degree of 0.4. Similar to all-2-DAP bilayers which were described in our previous work,^22^ the films adhered without additional adhesives or bonding agents via dynamic interfacial bonding. Holding the bilayer under 15% strain at 60 °C to develop a mismatch in the stress relaxation followed by the bilayer release resulted in bending toward the plasticized 2-DAP layer side (Figure 3b). The curvature increased with time in tension (*t*_t_), reflecting the progressively growing mismatch in the internal stress (Figure 3c), in agreement with simple beam bending theory (Supporting Information).

**Figure 3:**
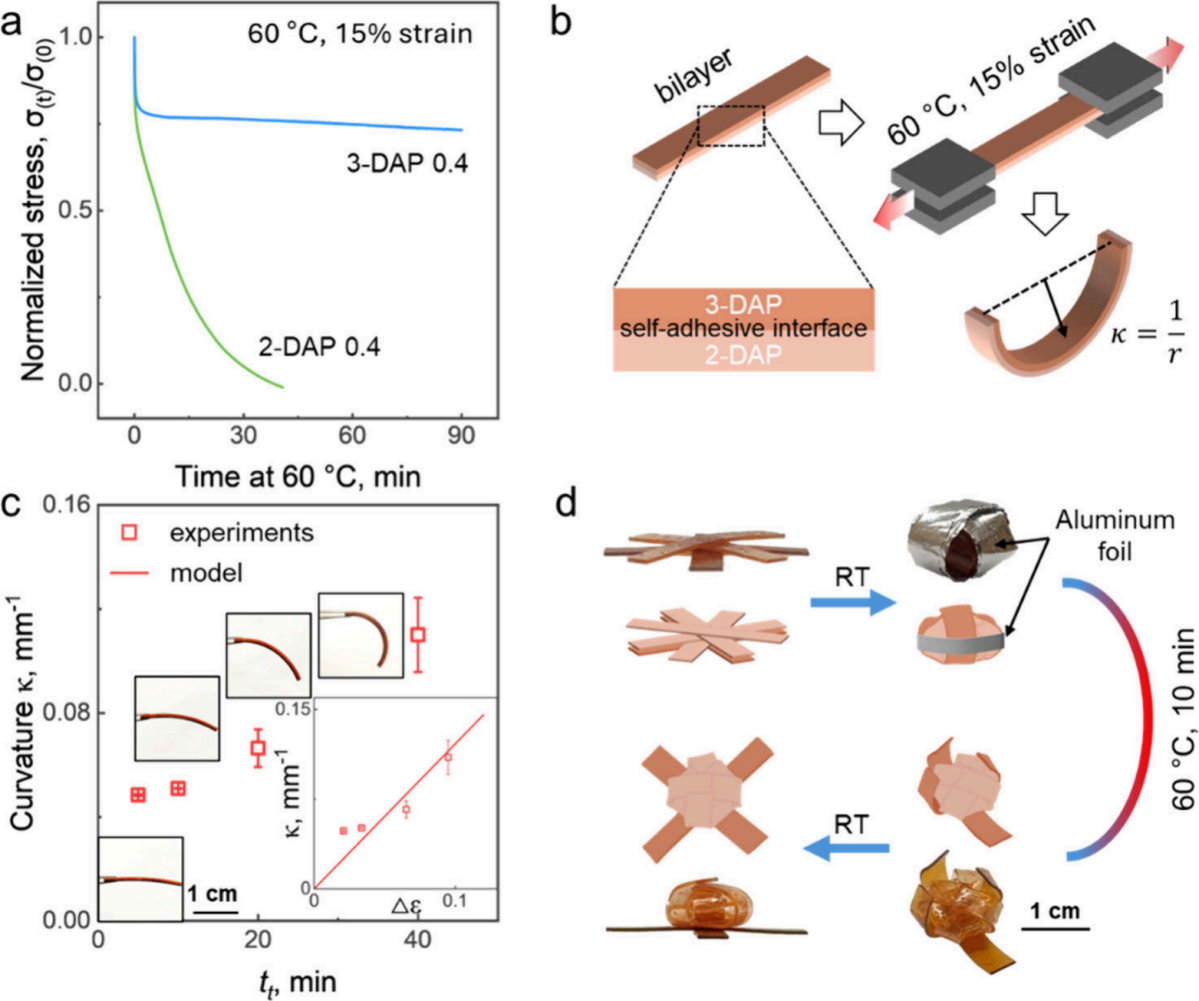
Relaxation behavior and shape morphing of 2- and 3-DAP networks. (a) The stress relaxation curves of 2-DAP 0.4 and 3-DAP 0.4 at 60 °C under 15% tensile strain. (b) Schematic illustration of the bilayer bending experiments, where self-adhered bilayers were held at 60 °C under 15% strain for time in tension, t_t_ and then released at room temperature. (c) Bending curvature (κ) of the shape-morphed bilayer vs *t*_t_. In the inset, experimental values of κ are compared with the predictions from the Simple Beam Bending Theory. (d) Schematic workflow along with the images of morphing of stacked 2- and 3-DAP films, demonstrating differential shape retention.

Building on this principle, a multilayer structure was assembled by radially stacking several 2-DAP and 3-DAP ribbons. The stacked object was compacted by wrapping with aluminum foil to apply a gentle mechanical constraint, held at 60 °C for 10 min and unwrapped to release the load under simultaneous cooling to room temperature. This resulted in a spatially programmed shape transformation (Figure 3d), in which 3-DAP films preserved their elasticity and recovered to their original flat shapes, while 2-DAP films maintained their curvature developed due to the network plasticization. This selective network plasticization resulted in the formation of a flower-like object which emerged due to the differences in the rate of dynamic bond exchange within the CAN materials which were constructed from the two furan regioisomers.

To map out the temperatures at which the networks can be shape-morphed through solid-state plasticity, we prepared flat ribbons made of individual 2-DAP or 3-DAP networks, curled and fixed them with aluminum foil to maintain their curled shape at room temperature, and then annealed them at elevated temperatures between 40 and 140 °C. After annealing for 10 min, the curled ribbons were cooled and aluminum foil removed. This procedure resulted in different degrees of the programmed curvature retention (Figure 4a). Figure 4b shows the retained shapes of 2- and 3-DAP ribbons along with the storage modulus–temperature curves. The distinct shape programming windows were correlated with the length of the rubber elasticity plateau for the two networks. For 3-DAP networks, shape morphing could be performed across a broad range of temperatures between ∼80 and 140 °C. Within this temperature range, the network underwent plasticization through partial DA bond dissociation and exchange, enabling permanent adaptation of a new curled shape. Below ∼80 °C, the curled ribbon elastically recovered after 10 min of morphing due to the network elasticity and lack of dynamicity of DA bonds. At 80 °C, only partial morphing of 3-DAP 0.4 was observed after 10 min; however, complete morphing was achieved after 30 min (Figure S7b). In contrast, 2-DAP networks had a much narrower and lower onset of temperature window (60–90 °C) for shape programming. Heating above this range led to a loss of structural integrity, consistent with a sharp decrease in storage modulus in the proximity of *T*_*rDA*_ of 120 °C. This low temperature stability significantly limits shape programmability of 2-DAP-based CANs.

**Figure 4:**
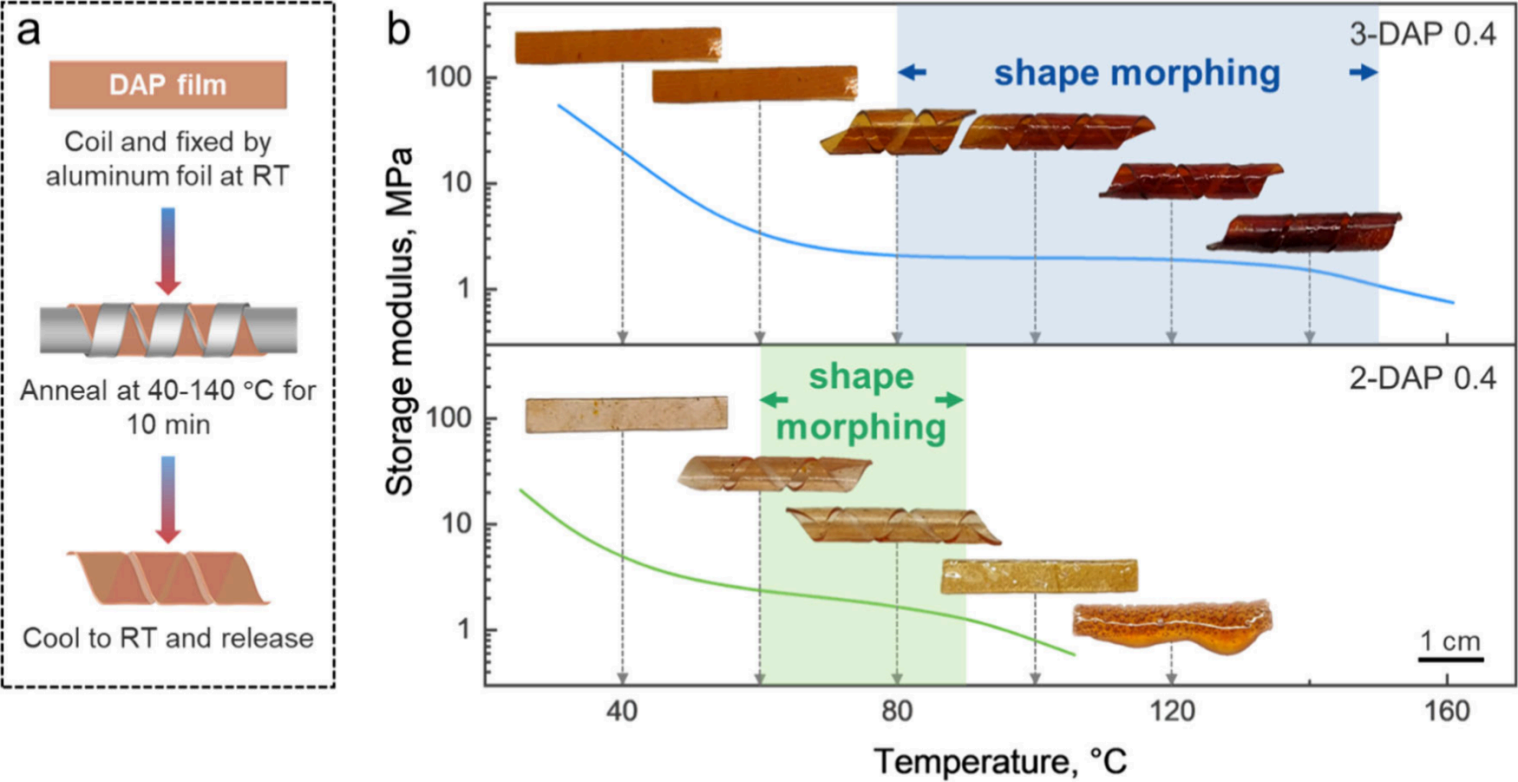
Single-material curling of 2- and 3-DAP 0.4 networks. (a) Schematic illustrating the steps for curling DAP ribbons. (b) Temperature dependence of the storage modulus of 2-DAP 0.4 and 3-DAP 0.4 networks with highlighted temperature windows for shape morphing. The gray dashed arrow denotes the annealing temperatures used to curl the DAP ribbons.

Taken together, our results illustrate how molecular-level substitutions can be translated into programmable functional responses in adaptive polymer materials. With a specific example of the chemical substituents in the furan ring, this work establishes regioisomerism as a powerful handle for controlling shapeability of soft materials and introduces elastomers based on 3-substituted furans as promising shape morphing materials. These elastomers, unlike the conventional materials based on 2-substituted furans which can plasticize uncontrollably at environmentally achievable temperatures (such as 60 °C), retain elasticity until much higher temperatures. The higher onset of network dissociation and a broader temperature window for shape morphing of the 3-substituted networks make them more practical reconfigurable materials for soft robotics and consumer goods. In addition, intrinsic self-adhesive behavior of DAP networks enables multimaterials’ with spatiotemporal control of shape morphing—a control that can be solely achieved due to the effect of regiochemistry on dynamic covalent reactions.

## Supplementary Material



## Notes

Experimental details, GPC and DSC data for prepolymers, evolution of the network *T*_*g*_ during temperature annealing, FTIR analysis of DA conversion, stress–strain curves, plots of elastic modulus and ultimate tensile strength vs degree of cross-linking, and stress relaxation curves (PDF)
