# Supplementary material for: Regiochemical Control of Shape Morphing in Diels–Alder Covalent Adaptable Networks
Source: ACS Macro Lett. 2025 Oct 1;14(10):1497–503. doi: 10.1021/acsmacrolett.5c00465 (PMC12548357; doi:10.1021/acsmacrolett.5c00465)
Supplement: Supplementary file 1 [file mz5c00465_si_001.pdf]

Supporting Information

Regiochemical Control of Shape Morphing in Diels-Alder Covalent Adaptable Networks

Yilei Zhao, Junho Moon, and Svetlana A. Sukhishvili*

Department of Materials Science and Engineering, Texas A&M University, College Station, TX 77843, USA

E-mail: svetlana@tamu.edu

Table of Contents

1. Experimental Section....3

1.1 Materials....3

1.2 Preparation of 3-substituted DAP network....3

2. Characterization....4

2.1 Gel Permeation Chromatography....4

2.2 Fourier Transform Infrared Spectroscopy (FTIR)....4

2.3 Differential Scanning Calorimetry Analysis....5

2.4 Dynamic Mechanical Analysis....5

3. Thermal annealing for complete conversion....6

4. Shape morphing....7

4.1 Bilayer bending experiments....7

4.2 Multi-layer morphing....8

4.3 Helical shape programming....8

Supporting Figures....9

References....15

1. Experimental Section

1.1 Materials

3-Furanmethanamine (3-substituted furan) was purchased from Angene Chemical. Neopentyl glycol diglycidyl ether (NGDE) and 1,1'-(methylenedi-4,1-phenylene)bismaleimide (BMI) were purchased from Sigma. Hydroquinone was purchased from ACROS Organics. Dichloromethane (DCM) and N,N-dimethylformamide (DMF) were obtained from VWR Chemicals. All the chemicals were used as received.

1.2 Preparation of 3-substituted DAP network

To synthesize the linear prepolymer, 3-substituted furan and NGDE were combined in a 1:1.9[1] molar ratio of amine-to-epoxide groups in a 20 mL vial. The mixture was degassed and purged with argon gas three to four times to remove dissolved air. The epoxide ring opening reaction with amine (Figure S1a) was carried out in a 100 ℃ oil bath under an argon atmosphere with continuous stirring for 16 hours, yielding a yellowish oligomeric liquid with an average molecular weight (M_w) of ~7000 g/mol, as determined by GPC (Figure S1b), and a glass transition temperature (T_g) of -14.7 °C, as determined by DSC (Figure S1c).

To prevent BMI self-polymerization, which typically occurs above 160 °C,[2] a solvent evaporation method was used to fabricate DAP network. A stoichiometric mixture of the prepolymer, BMI and hydroquinone (5 wt% relative to BMI) was dissolved in dichloromethane and stirred for 30 minutes. The obtained solution, with a concentration of ~150 mg/mL, was then cast onto a PTFE substrate and left to evaporate overnight at room temperature to form a dynamic

polymer network. These films were designated as DAP Φ_{BMI} (Φ_{BMI} = 0.2, 0.4, 0.6, 0.8 and 1.0), where Φ_{BMI} represents the molar ratio of maleimide to furan functional groups.

2. Characterization

2.1 Gel Permeation Chromatography tests

Gel permeation chromatography (GPC) was performed using a TOSOH EcoSEC 8320 system (Japan) to determine M_w and polydispersity index (PDI) of the prepolymer. Approximately 3-5 mg of the prepolymers was completely dissolved into 1 mL of DMF, followed by filtration through a 0.22 μm PTFE filter membrane to prevent column clogging. To compare the performance of 2-DAP and 3-DAP prepolymers, the effect of reaction temperature on the M_w and PDI of the 3-substituted prepolymer was first investigated (Figure S1b). For further study, 100 °C was selected as the optimal reaction temperature, where 3-substituted prepolymer achieved M_w and T_g comparable to those of 2-substituted prepolymer reported in our previous work.[1,3]

2.2 Fourier Transform Infrared Spectroscopy (FTIR) tests

To characterize the chemical structures of the monomers, linear prepolymers and the polymer networks, attenuated total reflectance Fourier-transform infrared spectroscopy (ATR-FTIR) was performed using a Bruker Tensor II spectrometer (USA) with an MCT detector. The spectra were recorded over the range of 4000-500 cm^{-1} with a resolution of 4 cm^{-1} (Figure S3).

The conversion of DA reaction, *i.e.*, the percentage of reacted furan corresponding to the amount of added BMI crosslinker, was also evaluated using FTIR. (i) All the spectra were baseline-corrected and normalized to the polymer backbone peak at 1096 cm^{-1}; (ii) The DA adduct peaks

at 1190 cm^{-1} for DAP networks with different Φ_{BMI} was integrated and normalized to the intensity of this peak in DAP 1.0 network.[3] The ratio of the peak areas represented the consumption of the furan moiety. (ⅲ) To quantify the conversion of DA reaction, the following equation is used:

$$Consumption\ of\ furan\ moiety = \frac{area\ of\ DAP\ \Phi_{BMI}\ at\ 1190\ cm^{-1}}{area\ of\ DAP\ 1.0\ at\ 1190\ cm^{-1}} \times 100\%$$

$$Conversion\ of\ DA\ reaction = \frac{Consumption\ of\ furan\ moiety}{Amount\ of\ added\ BMI} \times 100\%$$

2.3 Differential Scanning Calorimetry Analysis

Thermal analysis was conducted using a TA Instruments DSC 2500. The samples were scanned from -50 °C to 175 °C at a heating rate of 10 °C/min under a nitrogen atmosphere. The flow rate was 50 mL·min^{-1}. The glass transition temperature (T_g) was determined from the kink in the heat flow curves. The dissociation temperatures (T_{endo} and T_{exo}) were determined via deconvolution of the endothermic peaks related to the DA bond dissociation.

2.4 Dynamic Mechanical Analysis

To detect the mechanical properties of 3-DAP were evaluated using TA Instrument DMA850 system. Stress-strain, stress relaxation and temperature sweep tests were performed as described below. The stress-strain tests were conducted according to ASTM D638 standard using rectangular specimens (20 × 0.5 × 0.6 mm). The elastic modulus and ultimate tensile strength were calculated from the resulting curves. Each measurement was repeated three times. The stress relaxation tests were performed at temperatures ranging from 60 to 120 °C for 90 minutes at a constant strain of 15%. The initial force was set to 0.01 N, and data were collected at a sampling rate of 10 points

per second. The temperature sweep tests were conducted from 20 °C to 160 °C at a frequency of 1 Hz with a temperature increment of 10 °C. Storage modulus and loss tangent (*tan δ*) were obtained to assess thermal–mechanical behavior. For stress relaxation and temperature sweep tests, the sample dimensions were 30 × 0.5 × 0.6 mm.

3. Thermal annealing for complete conversion

To ensure complete DA crosslinking in 3-substituted furan-maleimide (3-DAP) networks, thermal annealing was required following the initial solvent-assisted mixing process. This was needed due to the high retro-DA (rDA) temperature of the 3-substituted adduct (~145 °C), which did not allow us to use the one-step melt processing strategy which was previously developed for 2-DAP networks. As-prepared 3-DAP samples typically remained partially crosslinked due to the limited chain mobility during solvent evaporation and the kinetic constraints of DA bond formation at ambient temperature. ATR-FTIR was used to monitor adduct formation. As shown in Figure S3, as-prepared 3-DAP networks displayed weak DA adduct bands (*i.e.*, the C–O–C stretch at 1190 cm^{-1}), whereas annealed samples exhibited stronger signals. These spectral changes confirm the increased adduct formation and improved network conversion upon annealing. The effect was more evident in the networks with higher crosslinking degrees (Fig. S3c), where steric congestion further slowed the reaction during solvent drying.

The effect of annealing on the network crosslinking was also tracked via changes in the network glass transition temperature, T_g. As shown in Fig. S2a, T_g of 3-DAP gradually increased with annealing time at 60 °C, reflecting both continued DA bond formation and stereoisomeric rearrangements. Direct analysis of the *endo* content during annealing using DSC (Fig. S5) revealed that within the first 4 h required for complete DA bond formation, only ~6 % of *endo* isomers were

converted to the *exo* form, while T_g increased by ~10 ℃ (Fig. S3a). Continued annealing up to 10 h resulted in an additional ~ 5 ℃ increase in T_g, accompanied by ~30 % loss of endo isomers. Thus, T_g growth during the first 4 h was dominated by enhanced DA bond formation, while longer annealing was accompanied by a stronger contribution of *endo*-to-*exo* isomerization. In contrast, 2-DAP samples prepared by a solvent-free technique showed no T_g shift during room-temperature aging, confirming complete conversion within 1 hour of the network synthesis. Upon annealing at 60 °C, 2-DAP did exhibit a minor T_g increase, which we attributed not to new bond formation, but to *endo*-to-*exo* isomerization as discussed in our prior work.[4] Taken together, these results establish thermal annealing as a critical step for achieving full conversion in 3-DAP systems prepared via solvent-assisted mixing. A 24-hour annealing period at 60 °C was sufficient to complete the network formation in 3-DAP networks without triggering undesired BMI self-polymerization.

4. Shape morphing

4.1 Bilayer bending experiments

The bending behavior of bilayer structures was analyzed based on the Simple Beam Bending Theory[5]. The bending curvature, κ (mm^{-1}), was calculated as a function of the elastic modulus E_i, strain mismatch $\Delta\varepsilon$ (%) and the thickness d_i (mm) of bilayer of each layer structure. The relationship is given by the following equation:

$$\kappa = \frac{6E_1'E_2'd_1d_2(d_1+d_2)\,(\eta_1\varepsilon_1-\eta_2\varepsilon_2)}{{E_1'}^2{d_1}^4+{E_2'}^2{d_2}^4+2E_1'E_2'd_1d_2(2{d_1}^2+2{d_2}^2+3d_1d_2)}$$

where $E_i' = \frac{E_i}{1-\nu_i^2}$, $\eta_i = 1 + \nu_i$, ν_i is Poisson's ratio (assumed to be 0.3 for each layer), i is the number of each layer. The equation can be simplified as:

$$\kappa = \frac{7.8 d_1 d_2 (d_1 + d_2)\Delta\varepsilon}{\frac{E_1}{E_2} {d_1}^4 + \frac{E_2}{E_1} {d_2}^4 + 2 d_1 d_2 (2{d_1}^2 + 2{d_2}^2 + 3 d_1 d_2)}$$

4.2 Multi-layer morphing

To demonstrate the different thermal behavior of 2- and 3-DAP, a flattened flower-shaped structure was constructed (Figure 3d). A total of six ribbons— *i.e.,* four pieces of 2-DAP 0.4 and two pieces of 3-DAP 0.4—were stacked radially to mimic the flower petals. Before stacking, these strips were cleaned thoroughly using acetone to remove surface contaminants and then gently pressed together at room temperature to trigger interfacial adhesion at the center.

After assembly, the stacked ribbons were manually bent into petal-like shapes and wrapped in the aluminum foil to temporarily maintain the desired structure during annealing. The construct was kept in an oven at 60 °C for 10 minutes, and then naturally cooled to room temperature. After release from the aluminum foil, the 3-DAP 0.4 "petals" flattened, while the 2-DAP 0.4 petals plasticized and retained their programmed new shape.

4.3 Helical shape programming

To investigate the shape-morphing behavior of the single-layer networks, 3-DAP and 2-DAP ribbons were fabricated with dimensions of 30 mm × 5 mm × 0.6 mm. Each ribbon was wrapped around a cylindrical metal rod with a diameter of 3 mm to introduce a helical shape and fixed by

aluminum foil at RT (Figure 4a). The wrapped samples were then thermally annealed in a temperature-controlled oven at constant temperatures ranging from 40 °C to 140 °C for 10 min.

Following the thermal treatment, the samples were allowed to cool naturally to room temperature while still constrained on the metal rod. Once fully cooled, the ribbons were carefully released, allowing them to undergo autonomous reconfiguration. The resulting spiral geometries were used to evaluate the efficiency of thermally induced morphing and temperature windows for shape morphing of 2-DAP and 3-DAP networks (Figure 4b).

Supporting Figures

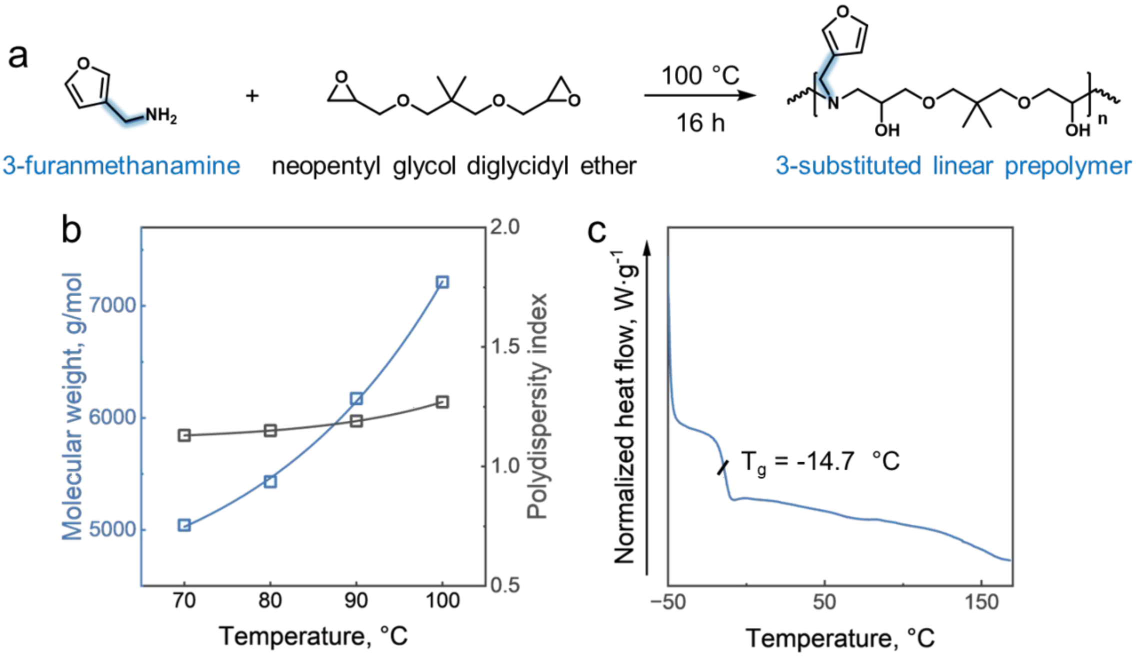

Figure S1. (a) Synthesis of linear prepolymer via epoxide ring-opening with amine. (b) The dependence of the average molecular weight and polydispersity of the prepolymers (as determined

by GPC) with a 1:1.9 molar ratio of the amine-to-epoxide groups on the reaction temperature. (c) DSC scan of the 3-sub linear prepolymer synthesized at 100 °C.

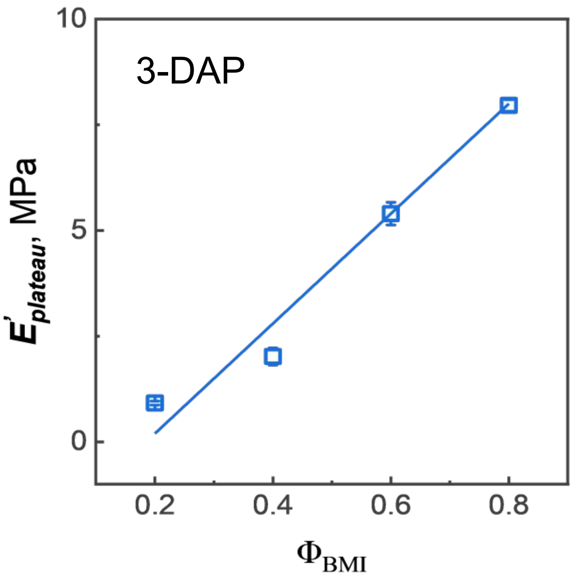

Figure S2. Rubbery-plateau storage modulus ($E'_{plateau}$) as a function of the maleimide-to-furan ratio (Φ_{BMI}) for 3-DAP networks. $E'_{plateau}$ was extracted from the rubbery plateau storage modulus measured by dynamic mechanical analysis (DMA). The linear scaling of $E'_{plateau}$ with Φ_{BMI} indicates that number of effective elastically active crosslinks increased linearly with BMI content, indicating high crosslinking efficiency.

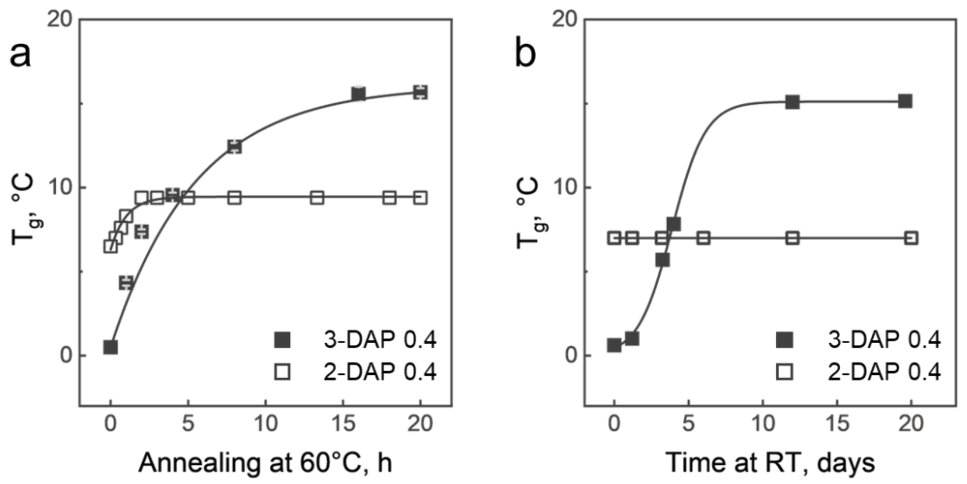

Figure S3. Time evolution of glass transition temperature of 2- and 3-substituted DAP 0.4 networks during annealing at 60 °C (a) and at room temperature (b).

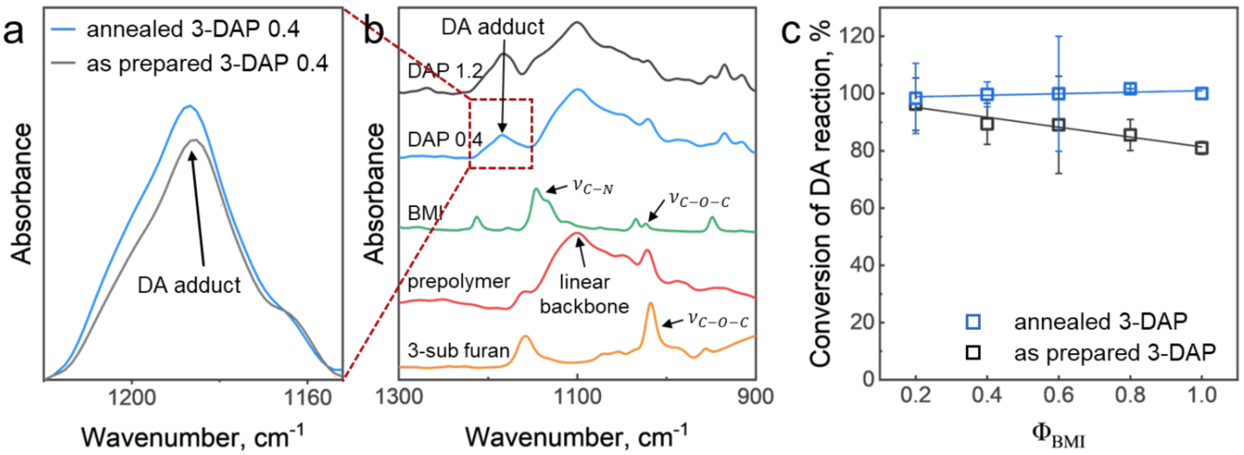

Figure S4. (a) An increase in intensity of the 1190 cm^{-1} peak associated with -C-O-C- stretching vibrations in the DA adduct of 3-DAP 0.4 network before and after 5-hr annealing at 60 °C. (b) The ATR-FTIR spectra of 3-DAP (Φ_{BMI} = 0.4 and 1.2) network and its components in the range from 900 to 1300 cm^{-1}, showing the -C-O-C- stretching vibrations of the furan ring at 1021 cm^{-1} and -C-N- and -C-N-C- stretching vibrations of BMI at 1146 cm^{-1} and 1023 cm^{-1}, respectively. (c) Conversion of DA reaction, before and after temperature annealing, as a function of Φ_{BMI} used for the synthesis of 3-DAP networks. The data are calculated by integrating the 1190 cm^{-1} DA adduct peak and normalizing it to the intensity of 3-DAP 1.0.[3]

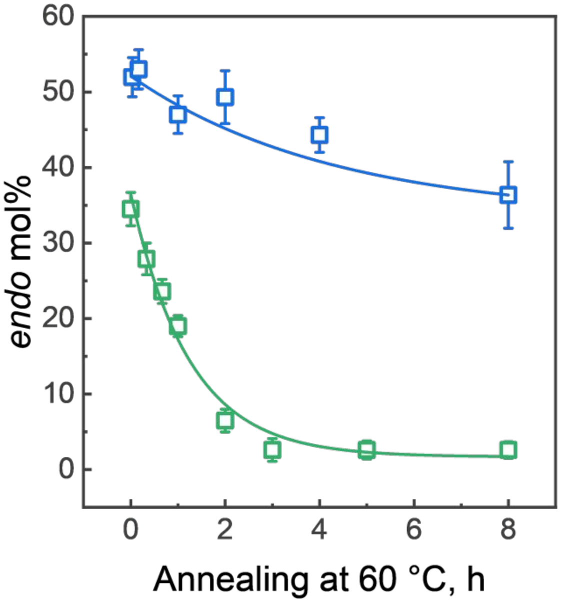

Figure S5. Time-dependent changes in the stereoisomeric composition (*endo* content) of DAP 0.4 networks during pre-annealing at 60 °C. *Endo* content was quantified by deconvoluting the thermal dissociation peaks in DSC thermograms, where the lower-temperature peak corresponds to the kinetically favored *endo* adduct and the higher-temperature peak corresponds to the thermodynamically favored *exo* adduct.

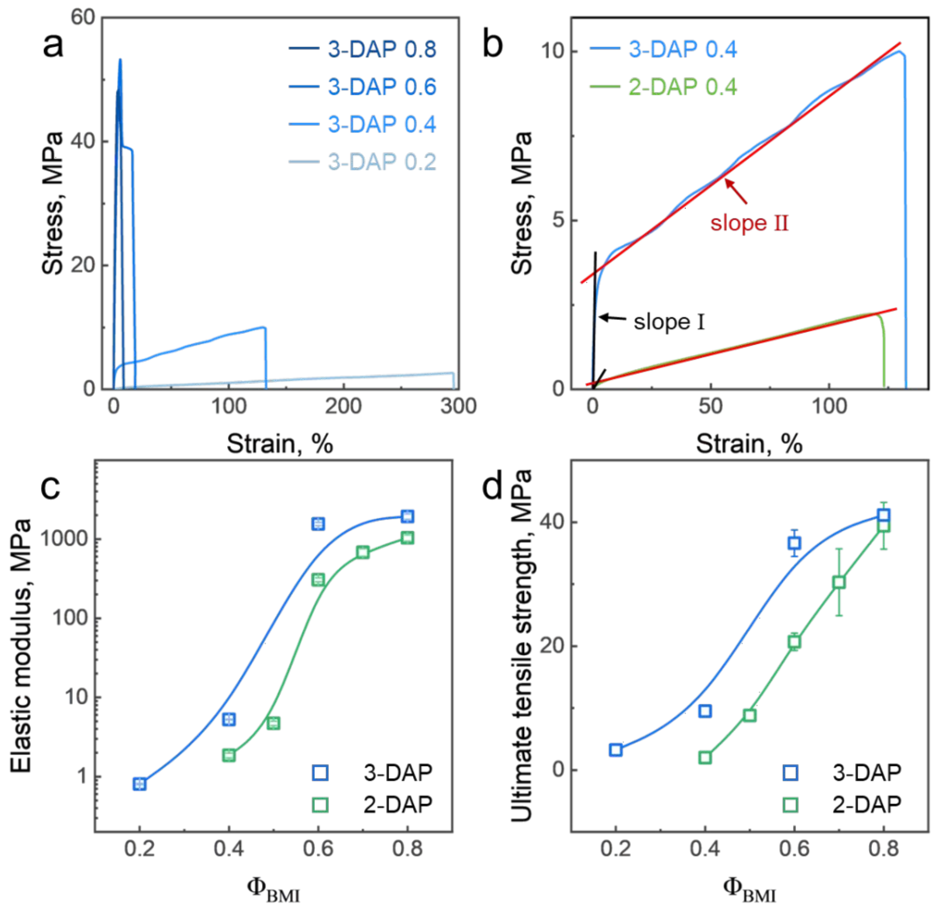

Figure S6. Mechanical properties of the 2-DAP and 3-DAP networks at 25 °C. (a) The stress-strain curves of 3-DAP networks with different crosslinking degrees. (b) A comparison of the stress-strain behavior of 2- and 3-DAP networks, which is divided into two distinct regions with different slopes. Slope Ⅰ is equivalent to Young's modulus with a 0.02% offset strain criterion, while slope Ⅱ is determined as the elastic modulus in a fully recoverable strain region. (c) Elastic modulus calculated from the slope in the region Ⅱ. (d) The ultimate tensile strength determined from the stress-strain curves in panel a. The elastic modulus and ultimate tensile strength of 2-DAP networks with different crosslinking degrees were calculated as described in our previous work (Figure 3c in Ref. 1).[1]

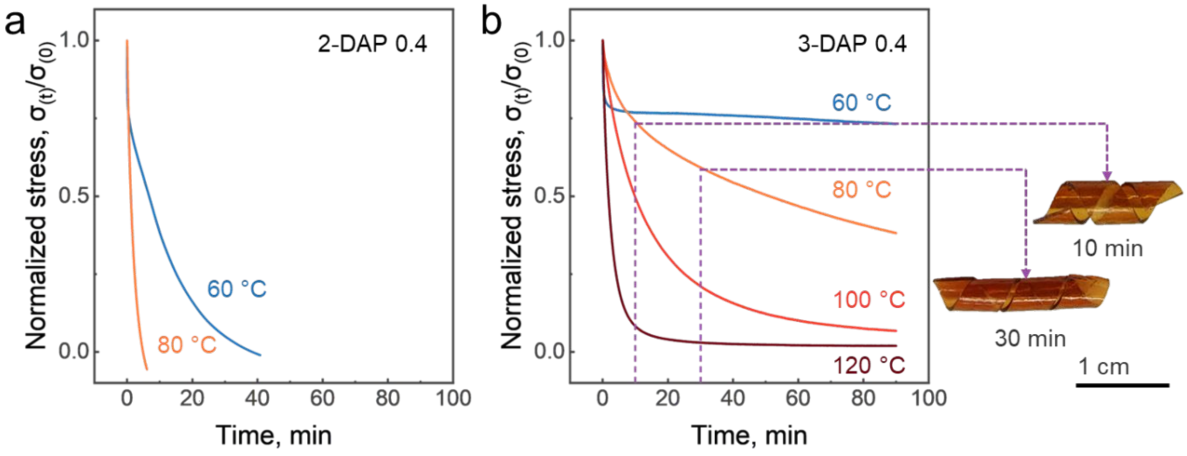

Figure S7. The stress relaxation curves of 2-DAP 0.4 (a) and 3-DAP 0.4 (b) networks at varied temperatures as measured by DMA after application of a 15% strain.

References

(1) Zhou, Q.; Gardea, F.; Sang, Z.; Lee, S.; Pharr, M.; Sukhishvili, S. A. A Tailorable Family of Elastomeric-to-Rigid, 3D Printable, Interbonding Polymer Networks. *Adv Funct Mater* **2020**, *30* (30).

(2) Pan, J. P.; Shiau, G. Y.; Lin, S. S.; Chen, K. M. Effect of Barbituric-Acid on the Self-Polymerization Reaction of Bismaleimides. *J Appl Polym Sci* **1992**, *45* (1), 103-109.

(3) Zhou, Q.; Sang, Z.; Rajagopalan, K. K.; Sliozberg, Y.; Gardea, F.; Sukhishvili, S. A. Thermodynamics and Stereochemistry of Diels-Alder Polymer Networks: Role of Crosslinker Flexibility and Crosslinking Density. *Macromolecules* **2021**, *54* (22), 10510-10519.

(4) Moon, J.; Sang, Z.; Rajagopalan, K. K.; Gardea, F.; Sukhishvili, S. Stereochemical Shape Morphing in Diels-Alder Polymer Networks. *Small* **2025**, *21* (2).

(5) Nikishkov, G. P. Curvature estimation for multilayer hinged structures with initial strains. *Journal of Applied Physics* **2003/10/15**, *94* (8).
